# Supplementary figures and images for: linus: Conveniently explore, share, and present large-scale biological trajectory data in a web browser
Source: PLoS Comput Biol. 2021 Nov 1;17(11):e1009503. doi: 10.1371/journal.pcbi.1009503 (PMC8584757; doi:10.1371/journal.pcbi.1009503)

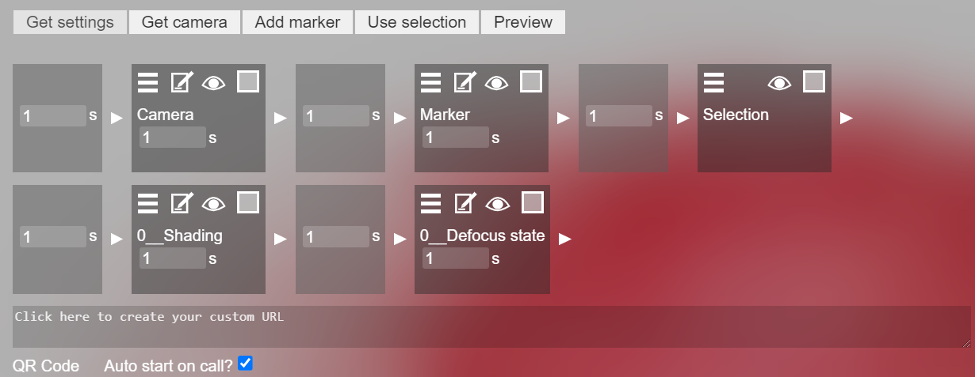

Supplement: S1 Fig — The tour actions can be organised by drag and drop (reading order: from left to right, top to bottom). Every action can be scheduled with a time delay with respect to the end of the previous action. Some actions use transitions (e.g. camera motions or the adjustment of numeric values) whose duration can be configured as well. Eventually, a URL or a QR code can be created. (TIFF) [file pcbi.1009503.s001.tiff]

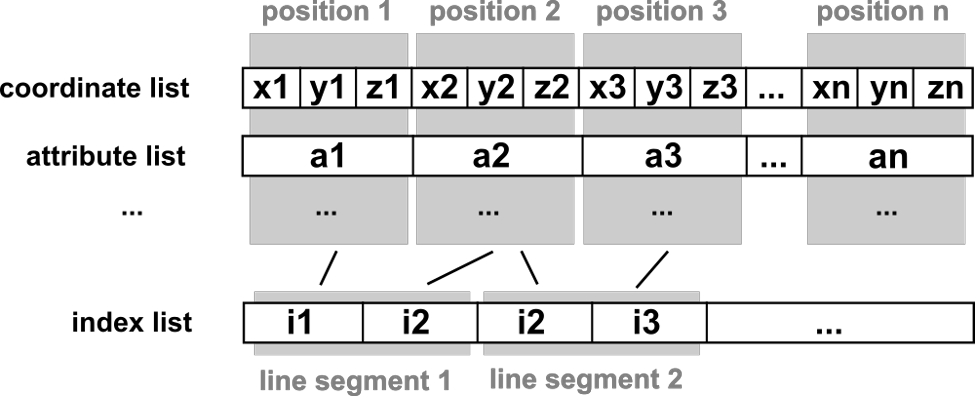

Supplement: S2 Fig — The coordinate list holds the x/y/z values for each supporting point of the trajectories. For each such point, an arbitrary number (only limited by the graphics card’s capabilities) of attributes can be stored. The attributes must be provided in the same order as the points. To create trajectories from the point set, an index list is provided as well. Each pair of indices describes one segment of a trajectory. The number of such segments is not restricted, as any point (and its respective attributes) can be used multiple times. (TIFF) [file pcbi.1009503.s002.tiff]

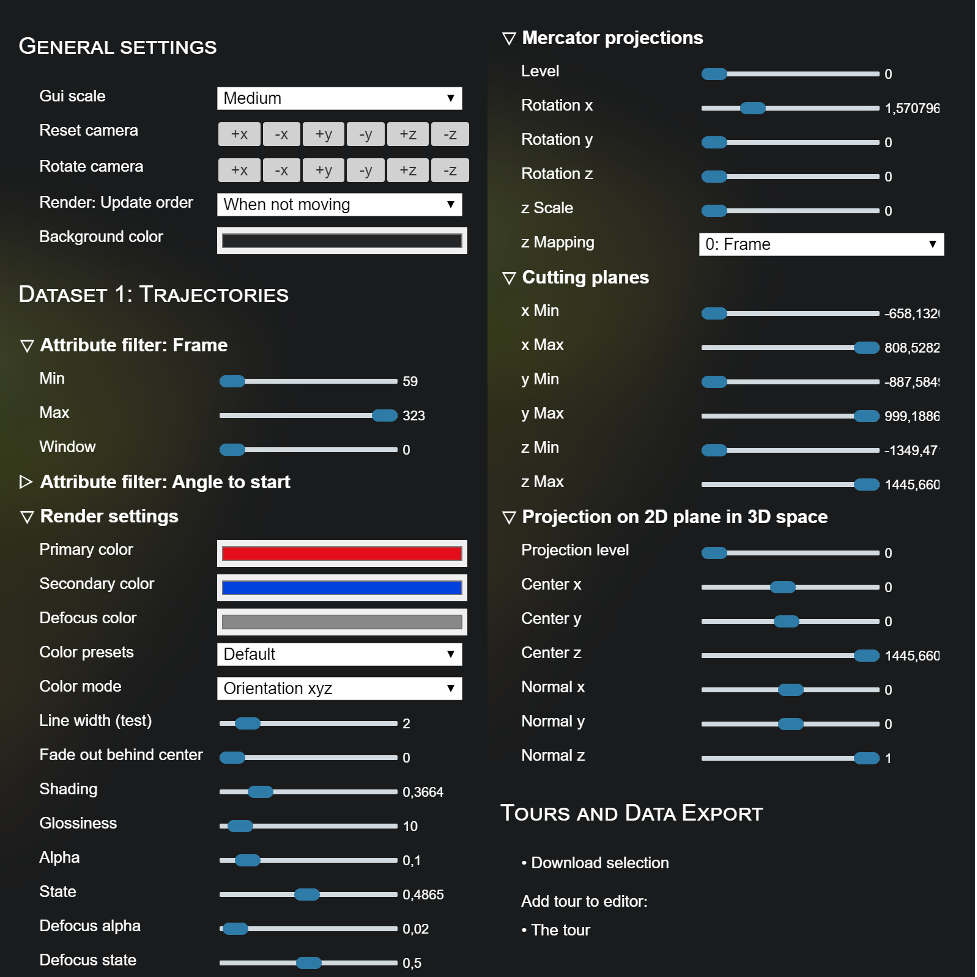

Supplement: S3 Fig — An overview of the different visualisation settings available to the user from the GUI (two screenshots merged). For explanations regarding different settings, see text or documentation at https://gitlab.com/imb-dev/linus. (TIFF) [file pcbi.1009503.s003.tiff]
