# Supplementary material for: linus: Conveniently explore, share, and present large-scale biological trajectory data in a web browser
Source: PLoS Comput Biol. 2021 Nov 1;17(11):e1009503. doi: 10.1371/journal.pcbi.1009503 (PMC8584757; doi:10.1371/journal.pcbi.1009503)
Supplement: S1 Text — (DOCX) [file pcbi.1009503.s005.docx]

# Supplementary Methods

## Design and Implementation

Our software consists conceptually of two parts: a Python-based preprocessing and a web-based visualisation tool. We aimed to move all static and computationally expensive adjustments to the preprocessor, whereas dynamic adjustments to tweak the visualisations are all be performed directly in the web browser later. After running the preprocessor, a folder containing HTML, CSS, and JavaScript files is created (called a visualisation packet). These files are opened directly or uploaded to a web server.

For more efficient computing, the preprocessing script uses established and optimised packages from python’s rich ecosystem, like NumPy and (Py)OpenCL. In particular, the edge bundling algorithm runs highly parallel on the graphics card and thus, about 10-100 times faster than a CPU-based calculation (with OpenCL-enabled hardware). However, only the creator of a *linus*-based visualisation package needs to run this preprocessor script. The target audience requires only a web browser to view and explore the data.

## Types of input data

We currently support different trajectory file types directly: TGMM [[1]](https://paperpile.com/c/M1ZZqK/qisc), biotracks [[2]](https://paperpile.com/c/M1ZZqK/meu9), SVF [[3]](https://paperpile.com/c/M1ZZqK/Xjeg), and custom CSV. Most formats are designed to store 3D coordinates plus a timestamp primarily, but no other custom data. However, *linus* supports additional numerical attributes that can then be used to filter or colour the trajectories accordingly. We, therefore, offer a generic CSV format which can be supplemented with custom numerical data: Each CSV file contains the data for a single trajectory, the first three columns represent the coordinates (x, y, z) and any further column is interpreted as another attribute. The columns are delimited by semicolons, and the number of columns must be identical for all CSV files. *linus* reads the first line of a CSV file by default as the header and uses this information to automatically name the respective properties in the user interface. The data converter script then expects a folder that exclusively contains CSV files as input.

## Implementation of data preprocessing

The trajectory data are then converted to a custom JSON format by our Python-based preprocessor. Python has the advantage of being executable on a wide range of operating systems and hardware. The preprocessor is used with a command-line interface or by calling the respective commands directly. The command-line interface is easier to use, and it covers the most common cases (e.g. visualising a dataset with custom attributes, and automatically adding an edge-bundled version). For more complex cases, e.g. visualising two datasets at once, or using multiple custom states of the data (e.g. custom projections), users can write their own Python script. We provide detailed and up-to-date documentation in our repository at <https://gitlab.com/imb-dev/linus>.

Time-consuming operations are implemented using NumPy, and the most demanding process (edge bundling) is handled by an OpenCL script, which increases calculation speed by 10-100 fold. All trajectories are resampled to equal length during the preprocessing step, enabling us to use NumPy’s fast matrix-based algorithms (we use $n*m$-matrices, storing $n$ trajectories with $m$ points in each trajectory). The resulting JSON file then contains a list of datasets. Each dataset holds a set of trajectories that optionally can be further organised into several states, for example, the original data and a projected version. At this point, all data are organised in the same structure as is required by WebGL (S2 Fig), which allows faster loading of the data in the next step.

## Implementation of the web-based tool

The visualisation part runs in web languages (HTML, JavaScript, CSS, WebGL). The JSON file containing the preprocessed data is directly loaded as an object by JavaScript. This part of the software copies the numeric arrays from the JSON file into WebGL's data buffers like the position buffer, index buffers, and attribute buffers. If a dataset contains more than one state (e.g. an original state and a projected state), these states are stored in additional attribute buffers. Depending on the provided data, we also adjust the shader source code dynamically. For example, we inject variables and specific statements into the shader source code before it is compiled by WebGL. With the dynamic creation of buffers as well as code statements and variables, we pre-build a shader program that is directly tailored to the properties of the respective data. As a result, rendering the data allows quick changes of the visualisation (e.g. colour mapping or projections) without the need for updating the datasets on the graphics card, which results in higher frame rates and smooth transitions compared to approaches where data is transformed offline.

In principle, *linus* supports an arbitrary number of attributes and states. However, practically this number is limited by the particular device’s abilities (i.e. its graphics card) and WebGL in general. Typically, we have eight attribute arrays on smartphones and sixteen or more on desktop computers. Our software requires four such attribute arrays for internal purposes, plus one more array for each state or attribute. Thus, for a dataset containing original data, bundled data and two custom attributes (that are shared between the states), we would need eight attribute buffers in total, which can still be managed by a smartphone. Visualising adding additional states or attributes requires devices with more capabilities, like a desktop computer.

## The graphical user interface (GUI)

The user interface (see Fig 1 and S3 Fig) consists of a general part that includes options to change the size of the GUI, the background colour, and camera controls. Furthermore, the user can choose how often the render order should be restored (see section "Technical limitations of web-based visualisations"). Additionally, several data-specific settings are shown, and this section is further divided into:

- *Filters* for each attribute to only show data within a defined range; if window is a positive value, it will be used to automatically display a range [min, min+window] (while max is ignored).
- *Render settings*, including colour mapping, shading, transparency, can be independently set for selected and unselected trajectories.
- *Mercator projection* plus rotations that are applied to the 3D positions before the 2D transformation, and mapping the "free" z component to attributes for 2D + feature plots (e.g. space-time trajectories).
- *Cutting planes* can be used to generate a generic 2D projection. Here, the projection plane can be defined by selecting a centre point and a normal direction. Everything above the projection plane is then mapped onto the plane.
- The last part of the GUI offers options to export selected trajectories and also shows a list of available tours. This list is used to start or to load a tour into the tour editor.

## Sharing visualisations and tours

As explained above, the user receives a self-contained package. This package can be opened with any web browser that supports WebGL and can be distributed in multiple ways: It can be locally shared (e.g. sent by email or copied using, e.g. a USB stick) or made easily accessible to a broad audience by uploading it to a web server (as done, e.g. on our companion website for this manuscript https://imb-dev.gitlab.io/linus-manuscript/).

The method of sharing the actual visualisation package also influences how an interactive tour can be distributed. In order to make a tour reproducible, they are internally represented by a textual list of actions. This script can be copied directly into the source code of the file main.html of the visualisation package. This method works both for server-based and file-based distribution of the package. If the visualisation package is hosted on a web server, the tours can also be shared simply with a custom URL and QR code that encodes a tour’s actions. However, the length of such tours is restricted: QR codes are limited in the amount of information they can store, and URLs are usually limited as well (but typically, this limit can be configured in the web server's settings). The commands for camera motion and parameter adjustment (e.g. changing the colour) are concise and only require a few bytes of the URL or QR code. In contrast, textual annotations and especially spatial selections require considerably more space. Thus, sharing a tour by QR codes or URLs usually works for tours without selections and without extensive text annotations.

## Specific considerations for virtual reality devices

The virtual reality mode works only when the visualisation package is hosted on a web server. Further, the way of navigation changes slightly because the head position takes over the task of the camera. For convenience, we introduce the possibility to adjust the height of the dataset and rotating the data horizontally. Inside the VR environment, no GUI is rendered. To allow controlling the GUI, the user can switch between "2D mode" and "VR mode" instantly.

## Export of trajectories

The user can select trajectories and download this selection. The download may take several minutes as the data must internally be converted into CSV format. The result is a zip folder containing one folder for each data set (usually a single folder), each containing a separate folder for each state of the data (e.g. "original" and "bundled"). Each trajectory is saved as a separate CSV file. It should be noted, however, that the user can only download the resampled trajectories and not trajectories in the raw (temporal or spatial) resolution before the data preprocessing.

## Screenshots and videos

At any time, the user can take screenshots and record videos with the respective buttons in the bottom left corner. Video recording requires an up-to-date Chrome-based browser (Chrome Version 52 or later; other browsers might support it as well but only with enabled experimental features). The output format is WebM, which is currently the only file type that can be directly saved from WebGL.

## Technical limitations of web-based visualisations

In order to offer the tool for a broader range of platforms, we decided to utilise WebGL 1.0. This web standard provides the feature set of OpenGL ES 2.0 (https://www.khronos.org/webgl/), which is limited compared to regular OpenGL versions. WebGL 1.0 is implemented by a wide range of browsers, such as Chrome Version 9, Firefox 4.0, Safari 8.0, iOS 8, Chrome mobile 30 (or newer, respectively).

The choice of a web-based visualisation solution brings some drawbacks. The amount of data that can be fluently visualised depends on the underlying hardware (smartphones: >2,000 trajectories, notebooks, and desktop computers: >10,000 trajectories). Another limitation is the reduced feature set which common web browsers offer regarding graphics card access: Compared to the API of OpenGL, the browser-based WebGL API offers fewer shader features. These restrictions lead to some limitations for the rendering process. A drawback of our rendering approach is that it creates artifacts related to the rendering order when we rotate the view (or actually the virtual camera in the 3D scene). Thus, we have to order the line fragments *offline* (i.e. not on the graphics card, but in JavaScript), which is a time-consuming process. To maintain high framerates, we only sort line fragments within a second after a user interaction has finished, leading to artifacts during camera motions (see below).

Our internal resorting procedure can require a noticeable amount of time (e.g. around 0.5 s for 10.000 trajectories). To ensure a fluent user experience, we use an adaptive strategy and only sort the data when the user stops moving the camera. This can lead to some visual artifacts during the rotation of the camera, but after stopping the motion, the correct rendering order is established quickly. For huge amounts of data, or for devices with low CPU performance (the sorting happens on the CPU, not on the GPU), it is also possible to completely disable the sorting. In that case, we shuffle the rendering order, which at least avoids distracting global patterns introduced by these artifacts. Furthermore, we cannot provide correct render order when rendering two datasets in the same view, and thus *linus* works best when only rendering one dataset at once.

When rendering a scene containing both trajectories and context, our application must render two different types of geometric primitives (lines and triangles) simultaneously. This can only be performed by two consecutive draw calls: the program first renders all triangles, and then we subsequently render the line segments. Since we need to support transparent rendering, we cannot rely on the z-buffer for determining the spatial order of the segments as this works only for non-transparent geometries (The z-buffer usually tells us if a segment should be drawn or not by checking if already another closer segment has been drawn that would cover the new segment). Thus, we use an alternative to the z-buffer: we sort the geometry first and render it starting with the most distant element. Step by step, we draw elements that are closer to the observer over more distant ones ensuring the correct depth ordering of elements. However, we cannot use this idea to compute the overlap between the set of triangles and the set of line segments since they are different types of primitives and, as such, require separate draw calls. As WebGL currently does not have a geometry shader, we cannot mix triangles and lines in one draw call. A consequence is that context can only be rendered as a background silhouette.

# References

1. [Amat F, Lemon W, Mossing DP, McDole K, Wan Y, Branson K, et al. Fast, accurate reconstruction of cell lineages from large-scale fluorescence microscopy data. Nat Methods. 2014;11: 951–958.](http://paperpile.com/b/M1ZZqK/qisc)

2. [Gonzalez-Beltran AN, Masuzzo P, Ampe C, Bakker G-J, Besson S, Eibl RH, et al. Community Standards for Open Cell Migration Data. bioRxiv. 2019. p. 803064. doi:](http://paperpile.com/b/M1ZZqK/meu9)[10.1101/803064](http://dx.doi.org/10.1101/803064)

3. [McDole K, Guignard L, Amat F, Berger A, Malandain G, Royer LA, et al. In Toto Imaging and Reconstruction of Post-Implantation Mouse Development at the Single-Cell Level. Cell. 2018;0. doi:](http://paperpile.com/b/M1ZZqK/Xjeg)[10.1016/j.cell.2018.09.031](http://dx.doi.org/10.1016/j.cell.2018.09.031)
