# Supplementary material for: linus: Conveniently explore, share, and present large-scale biological trajectory data in a web browser
Source: PLoS Comput Biol. 2021 Nov 1;17(11):e1009503. doi: 10.1371/journal.pcbi.1009503 (PMC8584757; doi:10.1371/journal.pcbi.1009503)
Supplement: Attachment — Submitted filename: response-to-review.pdf [file pcbi.1009503.s006.pdf]

Detailed response

Reviewer #1:

The authors present a new web toolkit for visualizing 3D data. The need for tools that can perform these functionalities is great in neuroscience right now. And they have built something that they seem to be using profitably. However, I have a few serious concerns, having been involved in writing software like this many times before.

1. Writing software like this is difficult, maintaining it is much more difficult. In the last 10 years there have probably been at least 10 published neuroscience visualization toolboxes developed, several of them for local clients, but also several of them browser based. Every single one failed unless it was developed by an institution (such as Google or Janelia). All the ones developed by labs died. The reason is that browser infrastructure and graduate students both change so far. Any toolkit will fail to work in a browser relatively quickly (say, ~2 years) without active developments, because the browsers change. And graduate students graduate, meaning that somebody else must take over the maintenance of the code. But graduate students like developing their own stuff, and also, they should develop their own stuff, because they need to if they want to graduate (they usually do). So, while I have no doubt that this tool is useful within the lab, I have serious evidence-based concerns about its utility more broadly. If (and this is a big if) the authors had a serious commitment (including financial commitment) for at least 5 years with a software engineer (rather than graduate student) who was committed to staying for 5 years, then I'd recommend the authors discuss the level of commitment extensively in the article, as tools without maintenance are not particularly useful.

We absolutely agree that maintenance is a general issue of scientific software that is often overlooked. This is also one of the core reasons why the Max Planck Institute for Human Cognitive and Brain Sciences has established a new Methods and Development Group for Neural Data Science and Statistical Computing headed by NS since January 2021. Developing advanced data visualisations, and building and maintaining scientific software is a core part of our support mission, and they are also central to the institute's agenda (https://www.cbs.mpg.de/methods-and-development-groups/neural-data-science-and-statistical-computing). Thus, as one of our central aims, we are committed to maintaining and developing this (and also related) software projects over the years to come with sustained support from the Neural Data Science group and the institute in general. Establishing a position for a research software engineer / scientific software developer is part of our concept to develop our institute's data science and open science agenda.

We also welcome the community to get involved and contribute their own ideas. As one example, we regularly organise hackathons (e.g. Brainhack) to connect people across the community (and across the globe) to foster contributions to open source tools for science. We do expect that regular updates could be necessary as the web-based visualisation frameworks (WebGL and Three.js) are subject to change. As suggested by (Romano and Moore 2020), we outlined a software management plan at our repository https://gitlab.com/imb-dev/linus/-/blob/master/CONTRIBUTING.md. We reference this CONTRIBUTING statement and explicitly invite contributions from the community now in the Availability and Future Directions section.

On a more general note, we want to stress that neuroscience was not the main application we had in mind when developing *linus*. Our trajectory visualisation targets a much broader range of applications that reach well beyond single neuron or tract imaging. We particularly want to address the audiences from cell and developmental biology and ecology, and we are convinced that there are much more use-cases we haven't yet explored.

While we agree that most software developed within labs does often not exist for long, we also believe that this is part of the process of scientific software development. We do not think that only large sites like Google or Janelia are the only credible sources for well maintained scientific software, tools like FIJI (https://fiji.sc) or the CAT toolbox (http://www.neuro.uni-jena.de/cat/) being just two examples from the field of biomedical imaging.

2. The tool actually does a few different things. First, it is a toolkit to enable visualization of fibers *at all*. Second, it is a set of features/functions/add-ons that enhance the visualization capabilities. It is actually the former that so worries me in my above comments. The latter could be generally useful, and even maintained long-term relatively easily, if they were plug-ins to already widely adopted software. For example, Neuroglancer (which was not cited) is currently used by hundreds of people. While my team is not actively developing NeuroGlancer, we were developing our own related tool until Neuroglancer came out, and then we switched. Fortunately, it seems there is lots of overlap between very Neuroglancer capabilities, and your functionality and code. For example, both are written primarily in TypeScript, and both have a Python API. While Neuroglancer was originally developed for electron microscopy data, people certainly visualize tracts using it as well. CATMAID can also render tracts in 3D. There are several other widely use web-browser based tools with overlapping functionality that are not mentioned at all. So, it is not clear the extent to which the functionality proposed here is novel/unique, nor it is clear how easy it would be to have features/plug-ins to other existing established tools, rather than building yet another neuroscience visualization engine. Here are some links to Neuroglancer that show some relevant functionality:

https://layer23.microns-explorer.org/

https://www.youtube.com/watch?v=zqjJkFmLauE

Much more in depth justification of a new tool rather than a fork/feature/add-on to NeuroGlancer, CATMAID, or other existing browser based web-visualizers would be required.

Thanks a lot for raising these points. We agree that our tool currently covers two different aspects: the import and the visualisation of trajectory data within a browser and a set of features to adapt and explore said visualisations. We have tried to make these aspects a bit clearer in the text now (see also the response to comment #4).

Regarding related tools, we did not claim to have invented browser-based visualisations of imaging data in this paper. We acknowledge that many great tools became available over the past years that build on WebGL and related techniques to support remote visualisation. We would like to clarify that we did not want to build yet another neuroscience visualisation engine. Our primary focus was creating an interactive and simple tool to collaboratively visualise and share (dense) trajectory data, something that, in our experience, is a common and important problem shared across all parts of biology (cell biology, neuroscience, ecology, animal behaviour, etc.) From our perspective, tools like CATMAID and Neuroglancer have a very different and specific focus: They have been developed as collaborative tools to work with volumetric neuroimaging data (in particular microcircuit reconstruction and annotation), and while they provide, e.g. WebGL-based neuronal morphology viewer and the option to share views via URL, these tools focus on use-cases that are quite different and complementary from ours.

Compared to these tools, we restrict our focus to one particular task (the visualisation of trajectories, but not images or annotations) but we have a different, much broader community in mind. Our aim is not so much to support the creation of accurate reconstructions of shapes from volumetric data, but to build lightweight, interactive visualisations of trajectory data of any kind (e.g. many tracks of moving objects) that prove to be challenging to visualise in static images otherwise. In particular, in developmental biology, there is a wealth of such data but tools for visualizing and exploring are scarce. We feel that this is very different from the tools described here and as such, would warrant a niche of its own. We have a more abstract level in mind: lines that can visually encode any type of trajectory. It's the versatility of *linus* with respect to data types that makes it unique.

We have adapted the introduction accordingly and outlined the distinction/justification for our tool in addition to related software projects like Neuroglancer or CATMAID and the motivation for building a general trajectory visualisation tool.

Furthermore, we would of course, be happy to add parts of our functionality to those tools as plug-ins wherever this would be beneficial for these communities. Apart from our own efforts to maintain and extend *linus* as a standalone project, we are also open to merging our ideas with existing open-source tools, so if this would be of interest to the community. We could very well image translating some functionality (say the tour editor or the QR code sharing) into plug-ins for existing projects such as

Neuroglancer. We have already integrated our interactive track bundling for example into the Sciview / Scenery plugin for FIJI [2,3].

3. Is it actually useful? There is no evaluation of its practical utility. Who currently uses it? Why? Is there empirical data one can point to? I found it difficult to control and navigate, and I did not understand the controls.

The value of a tool for the community will ultimately be judged by the community. We have sketched a range of applications that we and several collaborators found the tool to be very useful so far. With this publication, we want to increase the visibility of our tool so people can start using it and give us feedback on how to improve it further.

We have got a lot of positive feedback from the computational cell biology, developmental biology and neuroscience community, as reflected in the examples we have shown here. However, we see a much larger range of applications. We, unfortunately, have not conducted any kind of user study yet, but we would be happy to include the empirical analysis in a follow-up project.

We particularly thank the reviewer for pointing us to the difficulties with using the controls of our tool.We tried to improve the documentation considerably. We have revised our documentation online (https://gitlab.com/imb-dev/linus/-/blob/master/readme.md). Please note that some of the names for controls in the GUI our tool are not fixed as *linus* creates these controls in a generic way from the data attributes given in the input data (e.g. the *Attribute Filter*s that allow users to filter for subsets of any feature/column in the original data frame). Thus, the naming of these controls is generated automatically from the data input. Depending on the data's complexity, the number of attributes and filter options might indeed be overwhelming for the end-user (Group A mentioned in comment #2). In that case, the data presenter (Group B) could design a tour that guides the user through the data and hides the controls, which should avoid any confusion.

To make the process of creating the visualisation as clear as possible, we additionally provide a step-by-step guide now showing how to get from input data to the final visualization for a concrete non-trivial example.

4. There are 3 potential target audiences:

A. People who simply use the website to visualize some data. They need to understand user features, but nothing else.

B. Moderators: these people need to be able to deploy the code as well, ingest data, etc., and therefore understand more of the guts of the code.

C. Maintainers: these people need to be able to modify the code itself to add functionality, etc.

It is not clear to me exactly which of these audiences this manuscript is written for, it feels like it fluctuates between these three groups. Please make it clear to whom you are writing for any given paragraph, so it is clear.

Thanks a lot for pointing out this distinction. This distinction into three different audiences is an excellent suggestion. We agree that the presentation needed to be optimised in this respect. Also, following (Romano and Moore 2020), our major aim was to address group A in the main text (in particular in the Results). Group B is addressed in the *Design and Implementation* and the *Supplementary Method*s parts. For group C we refer to the Documentation in our online repository. We have revised the text accordingly with this perspective in mind, and hopefully, we could make it clearer for each part whom we are addressing here. In particular, we have moved the more technical part from the *Design and Implementation* section into the *Supplementary Methods*. We further add an explicit statement about the structure of the text to the end of the *Introduction* (We indicated the text that has been moved in red in the text to distinguish it from revised or added text in blue).

Reviewer #2:

J. Waschke et al. present a visualisation tool to investigate two- and three-dimensional trajectory data. The easy-to-use application allows to examine trajectories from all angles, modify the visual representation and select data subdomains for more in-depth examination in a web-based interface. The authors nicely place their tool in the context of a growing amount of tracking data and interdisciplinary research that would benefit from quick sharing of results such as visualizations.

Linus is a very useful application and many scientists will profit from this easy-to-use tool. The user interface is well designed, with beautiful visualizations and an intuitive workflow. The paper is well written and easy to understand. The authors clearly state which problems Linus is intended to solve. The documentation of the github repository contains all the relevant details and guides the user well. We found linus easy to apply from the git-repository and it worked as described on Mac on our own data. We particularly like the idea of shareable QR codes to switch between devices seamlessly. Finally, besides sharing visualizations with collaborators, the tool comes in handy to produce beautiful figures for manuscripts and talks.

We strongly support the publication of the tool and suggest addressing the issues below.

* Issues:

** Data handling:

- Data import should be more flexible. From a TrackMate .csv file we got the error “ValueError: could not convert string to float: '88.862;113.354;40''. We solved this by using a comma (“,”) instead of a (“;”) as a separator and converting the numbers. As this might be a recurring error for other users the authors should make the data loader more flexible and robust and thereby easier to use.

Thanks a lot for pointing us to this bug. We have found and fixed the issue: The CSV importer had an option to set the separator character (--csvSep, see the README at https://gitlab.com/imb-dev/linus). However, it seems this parameter was not read correctly. In the current version, this import option works as expected.

- We were not able to visualize multiple tracks contained in one .csv file as generated from TrackMate (e.g. with columns: Label, ID, TRACK_ID, QUALITY ,POSITION_X, POSITION_Y, POSITION_Z, POSITION_T ,FRAME). Can you direct the user with a few how tos / a video tutorial for the major use cases / data formats?

Thanks a lot for suggesting this extension. Indeed, this was not part of our initial assumptions about the data formats. The importer originally expected separate files for each track. To make the import more robust and generalisable, we have added the *--csvIdColumn* option to explicitly read the ID column from the CSV file, so multiple tracks per file can be imported now. We have updated our documentation at https://gitlab.com/imb-dev/linus accordingly. To make it easier for the community, we have now added the *-trackmate* option to directly import data from TrackMate. We also provide an extended tutorial that shows a typical use-case (the mouse embryo data from (McDole et al. 2018)) from data import to preparing the final visualisation.

Please add examples of files that can be imported and use cases to the github, which the user can follow. This would be very helpful.

Thanks a lot for this suggestion. As outlined above, we have added a step-by-step tutorial. We will also add further examples to our online documentation.

** The tool:

- Selecting and highlighting single tracks would be extremely useful.

Thanks a lot for suggesting this feature. We have added this feature so the user can hold ‘s’ and click at the desired track to create a tiny selection window around the current mouse position (otherwise, we found the selection to be too complicated). With more user feedback, we will continue to improve this functionality.

- Highlighting track directions between timepoints (in polar, spherical or cartesian coordinates) would be great, so one can easily explore movement patterns.

Thanks a lot for pointing us to this feature. Linus supports visualisation of the local 3D and 2D direction of a track using a generic encoding of the (dx, dy, dz) values to RGB (Setting *Color mode* to *orientation xyz* in the GUI). We show this now explicitly in Fig. 1 and also in Fig. 2d and 3a, respectively. For spherical coordinates and Mercator projections, the user can also select a colour mapping that shows longitude and latitude by choosing *orientation lon/lat*.

Additionally, we now have added the *Opacity mode* option to do a local alpha mapping that gradually fades out tracks in the past. In a variety of scenarios, this can be used to further enhance the visual highlighting of local movement directions.

We mention these options now in the main text and added them to the online documentation.

- Pls comment: How is the software going to be sustained?

Thanks for raising this important question relating to the first comment by reviewer 1. We refer to our answer there for the respective details.

* Minor issues:

** Wording:

- We suggest to use ‘often’ only once in the first sentences of abstract and into

Good point. We have changed that.

- Not clear what is meant by ‘camera’ when first used on page 8(? please add page numbers)

We have introduced the term camera now. Note that this part has been moved to the more technical *Supplementary Methods* part. We have also added page numbers in the revised manuscript.

- British vs American english: e.g. color vs colour, visualization vs visualisation

Thanks for pointing out the problems. We have adapted the main text to British English.

- Typo in Fig. 2 caption: “(d) The user can further create subselections of the tracks in space using cutting planes or refinable spatial selections.” Should be selections at the end of the sentence?

Thanks a lot. This mistake has been fixed.

- Type in Fig 3: ‘tracts’

This is actually the better term here for the white matter connectivity in the brain.

- Fig 3a: either ‘ant migration’ or ‘animal tracks’ we suggest.

We have adapted the figure accordingly.

** Structure:

- Last sentences of the intro ("We began…") feel a bit misplaced. Use the zebrafish example as the motivation?

We have rephrased the sentence.

The authors mixed up subfigures in Figure 3: (e) and (f) should be exchanged with each other and changed to (c) and (d).

Thanks a lot for finding this issue. It is fixed now.

- "Exemplary visualizations are available by scanning the QR codes in Fig.1" - It should be Fig. 3 here

We have fixed this error.

paragraph 'We tested linus…' is misplaced in 'Availability and future directions' we believe. Better separate into two sub chapters

Thanks a lot. We have now moved this part to the last results subchapter, where we felt it would fit better.

** Presentation:

- Fig 1b looks great, also for a cover, but maybe change to an example that reminds the reader less of toupee? ;-)

Thanks a lot for this suggestion. We have created an alternative version of 1b which we hope looks less like a toupee now (or now it looks at least like a quite colourful one). At the same time, the updated figure also highlights the encoding of local orientation into RGB values and shows the effect of bundling a bit better, in our opinion. Thanks a lot!

- Caption Fig. 1: Pls describe the steps in words, one by one

We have extended and revised the caption of Fig.1 to explain the different steps.

move 'Furthermore, we cannot provide…" sentence to a "Limitations of the approach" paragraph?

We moved this part to the (renamed) *Technical limitations of web-based visualisations* section of the *Supplementary Methods.*

- Fig 2: Not clear to me what the thin gray lines mean. Am I supposed to read from left to right? c to b?

The grey lines indicate the filtering of the data with respect to different data dimensions/features. The top part of Figure 2b shows the full dataset, and the bottom part indicates filtering for different time windows and c) shows filtering for two different ranges of a cell marker. We tried to make it clearer by adapting the caption of Fig.2 accordingly.

References

1. Romano JD, Moore JH. Ten simple rules for writing a paper about scientific software. PLoS Comput Biol. 2020;16: e1008390. doi:10.1371/journal.pcbi.1008390

2. Günther U, Pietzsch T, Gupta A, Harrington KIS, Tomancak P, Gumhold S, et al. scenery: Flexible Virtual Reality Visualization on the Java VM. arXiv [cs.GR]. 2019. Available: http://arxiv.org/abs/1906.06726

3. Günther U, Harrington KIS. Tales from the Trenches: Developing sciview, a new 3D viewer for the ImageJ community. arXiv [cs.GR]. 2020. Available: http://arxiv.org/abs/2004.11897
